# Dietary aspects related to health and obesity in Williams syndrome, Down syndrome, and Prader–Willi syndrome

**DOI:** 10.3402/fnr.v59.25487

**Published:** 2015-02-03

**Authors:** Marianne Nordstrøm, Benedicte Paus, Lene F. Andersen, Svein Olav Kolset

**Affiliations:** 1 Frambu Resource Centre for Rare Disorders, Siggerud, Norway; 2 Department of Nutrition, Institute of Basic Medical Sciences, University of Oslo, Oslo, Norway; 3 Institute of Clinical Medicine, University of Oslo, Oslo, Norway; 4 Department of Medical Genetics, Oslo University Hospital, Oslo, Norway

**Keywords:** diet, carotenoids, omega-3 fatty acids, obesity, intellectual disability, autonomy, developmental disability, living arrangements

## Abstract

**Background:**

Dietary aspects that might contribute to development of obesity and secondary conditions are not well documented in genetic subgroups associated with intellectual disability.

**Objective:**

To describe the intake frequencies of selected foods in participants with Prader–Willi syndrome (PWS), Down syndrome (DS), and Williams syndrome (WS), and investigate the association with body mass index (BMI). To explore food-related autonomy and intake frequencies among persons with DS in different living arrangements.

**Methods:**

Self-reported intake frequencies and measurement of plasma carotenoids and erythrocyte content of omega-3 fatty acids (FAs) were investigated in persons aged 16–42 years, with WS (*n*=21), DS (*n*=40), and PWS (*n*=20).

**Results:**

A larger proportion of participants with PWS showed high-frequency intake of fruits (*p*=0.012) and vegetables (*p*=0.004), and had higher plasma carotenoids (*p*<0.001) compared to participants with DS and WS. Furthermore, a larger proportion of participants with WS were low-frequency consumers of fish (*p*=0.005), less likely to use omega-3 FA supplements (*p*=0.023), and had reduced erythrocyte concentrations of long-chain omega-3 FAs (*p*<0.001), compared to participants with PWS and DS. In DS, BMI was negatively associated with plasma carotenoids. Increased proportions of participants living in communities showed high-frequency intake of precooked meals (*p*=0.030), and a tendency toward high-frequency consumption of soft drinks (*p*=0.079), when compared to peers living with relatives. Participants in community residences were also more likely to participate frequently in food-related decisions and preparations.

**Conclusions:**

Persons with WS had a less-favorable dietary pattern when compared to persons with PWS. A larger proportion of persons living in communities frequently consumed precooked meals and showed a tendency of high-frequency soft drink consumption. Otherwise, their intake frequencies of the investigated foods were similar to those living with relatives, but they participated more frequently in decisions and preparations of foods.

Currently, individuals with intellectual disability (ID) enjoy an increased life expectancy (1, 2) and experience increased integration in communities combined with focus on normalization and autonomy (3). Even so, persons with ID living in community residences have a high risk of developing obesity and secondary conditions related to poor lifestyle (4–7). In Norway all persons with ID are offered community residence with support when moving from their parental home. Support is financed by the local municipality, and is provided based on assessment of individual needs. This is a general national policy and provides an opportunity for descriptive studies of lifestyle-related factors in adult persons with ID living in community residences and also for comparisons between different genetic subgroups, and to those living with relatives.

A diet rich in fruit and vegetables and regular consumption of fish and omega-3 fatty acids (FAs) of marine origin are associated with several favorable health outcomes (8–11). In Norway, dietary guidelines to promote public health and prevent chronic diseases recommend a mainly plant-based diet rich in fruits and vegetables. Furthermore, use of long-chain omega-3 FAs supplementation is recommended to those with intake frequency of fish below two to three times a week (12). Suboptimal intakes of fruit and vegetables has been documented in the general adult Norwegian population, where about 35–40% meet the fruit intake recommendation and about 15% meet the recommendation of vegetable intake (13). Use of omega-3 FAs supplementation is common with daily use described in about 45% of the population (14). Previous studies of dietary intakes in adult persons with ID, have found low intakes of fruit and vegetables (6, 15–18), low intake frequency of fish (17), and low intakes of polyunsaturated FAs (19).

Sugar-sweetened soft drinks are important contributors to refined sugar intake (13), and the intake of soft drinks has been associated with increased energy intake and increased body weight (20, 21). Precooked meals consist of meals ready to eat or meals that only require heating. These meals come in a variety of subtypes and with variation in package size; both single portions and multi portions size are sold in grocery stores in Norway. They are often energy dense, with high fat and salt content, and often low in vegetables and fiber. Regular consumption of convenience food has been reported in adults with ID living in community residences (22). The consumption of precooked meals together with soft drinks is an important topic to investigate in groups of persons with ID, because of their possible influence on the risk of developing obesity, and the quality of diet (23).

Previous studies addressing dietary intakes in groups of adults with ID have mainly used food frequency questionnaires, food diaries maintained by proxy reporters, or combinations of proxy- and self-reported methods (6, 15, 16, 19). However, none of these dietary assessment methods have been validated for use in groups of adults with ID (24). Furthermore, the use of proxy reporters is inappropriate in persons with ID living mostly independently and receiving little support. Additional use of biomarkers therefore has the potential to objectively supplement the information generated from other dietary assessment techniques (25, 26).

Carotenoids are natural pigments found in fruits and vegetables that humans are unable to synthesize. Plant foods are therefore the primary source of carotenoids for humans. Six carotenoids, lycopene, lutein, zeaxanthin, β-cryptoxanthin, α-carotene, and β-carotene, can be found within a significant concentration range in plasma and can be used as biomarkers for fruit and vegetable consumption (27–29). FAs from the diet are incorporated into erythrocyte membranes (30). Erythrocytes have a lifespan of 120 days and lack the enzymes needed for FA metabolism; therefore, they are regarded as a reliable biomarker reflecting an individual's FA intake over the last several weeks. However, FAs not synthesized in humans, such as long-chain omega-3 FAs of marine origin, have the highest correlation with intake (31–33).

Williams syndrome (WS), Down syndrome (DS), and Prader–Willi syndrome (PWS) are genetic conditions mainly associated with mild-to-moderate ID, although a wider range in cognitive abilities can be observed. WS is caused by a deletion of the 7q11.23 chromosomal region (34). DS is due to the presence of an extra copy of a major portion of chromosome 21 (35), whereas PWS is caused by a lack of expression of paternally inherited genes in the 15q11-q13 chromosomal region due to deletion, maternal disomy 15, or an imprinting defect (36). Molecular diagnoses are available for all three conditions; however, diagnosis in some patients is based on clinical manifestations alone. PWS and DS are described to be associated with increased risk of obesity (6, 19, 36, 37), but this has not been described in any detail in relation to WS. In PWS the high risk of morbid obesity is linked to hyperphagia (38), reduced energy expenditure (39), and the obsessive behavioral profile of the syndrome (40). A low-energy diet combined with rigorous food supervision and restricted access to food is recommended management for the prevention of life-threatening obesity in this group (41–43). In DS reduced physical activity (44, 45), reduced basal metabolic rate (46), and increased risk of subclinical hypothyroidism are described as underlying features of the high obesity risk (47).

Persons with ID are a heterogeneous group, and to date few previous studies have included a description of dietary aspects among community-dwelling individuals in relation to diagnosis-specific subgroups. Furthermore, although high risk of obesity is described for persons with mild-to-moderate ID living in community residences (4, 5), dietary intakes and food-related autonomy in relation to weight status and changes in these aspects when moving from parental homes to community residences with support have previously not been investigated to any great extent. Therefore, in the present study, we aim to describe and compare the proportions with low and high intake frequencies of fruits, fruit juice, and vegetables; fish and omega-3 supplements; soft drinks and precooked meals; and biomarkers, and explore this in relation to weight status in individuals with DS, WS, and PWS. Moreover, the second aim was to explore possible differences in degree of food-related autonomy and proportion with low and high intake frequencies of the selected foods, between individuals in community residence with support and individuals living with relatives. In this second part of the study, we focused on persons with DS only.

## Methods

### Study sample

Participants were recruited from all over Norway using information circulated in collaboration with the following nationwide patient organizations: The Norwegian Association for Persons with Developmental Disabilities, The National Association for Prader–Willi syndrome, The Norwegian Association for Williams’ Syndrome, and The Norwegian Network for Down Syndrome. In addition, information on the study and consent forms were posted on a study-specific website. The inclusion criteria were diagnosis with DS, WS, or PWS verified by standardized clinical methods (48, 49) or by laboratory genetic testing; age 16–45 years; and returned consent forms signed by the participant and legal guardian or parent. In total, 104 returned a signed consent forms. Four participants with PWS, three participants with DS, and one participant with WS dropped out of the study, so a total of 96 participants took part in the data collection. In the data analyses, a total of 81 participants were included, after elimination of nine participants due to negative results from laboratory genetic testing and for not fulfilling the clinical diagnostic criteria. Furthermore, two persons with PWS and four persons with WS living with relatives were excluded, as they were too few to be included in a comparison with diagnose specific peers living in communities. For this reason, only participants with DS were included in the analysis in which different living arrangements were compared. Ethical approval for the study was granted by the Regional Committee for Medical and Health Research Ethics, Southeast Region.

### Collection of data

Data were collected during courses held at a national reference center for rare disorders in Norway. Participants attended the courses together with a parent or employed caregiver. All data collection was adapted to and performed in a manner to promote the participants themselves to be active and the main informants. An electronic questionnaire was used to collect demographic data (with the exception of weight and height): information about celiac disease diagnosis, hypothyroidism, and thyroid hormones substitution therapy; information on the intake frequency of fruits, vegetables, fruit juice, fish (including fish eaten both in hot and cold meals), soft drinks, and precooked meals; use of omega-3 supplementation; and degree of participation in decision-making about food and active preparation of food (Questback, Oslo, Norway). The participants, together with a parent/caregiver, used a computer to fill out the questionnaire during the courses. The instructed role of the parent/caregiver was to record the participant's response, and not themselves to be the main respondent. The food frequencies used were as follows: less than once a week, one to three times a week, four to six times a week, each day, and several times a day. These categories were later collapsed into two categories: three times a week or less, and four times a week or more, representing low- and high-frequency intake, respectively. Furthermore, we examined the proportion of daily fruit, vegetables, fish, and soft drink consumption, and the proportion of fish intake less than once a week. Information on daily use of omega-3 supplementation was collected based on ‘yes’ or ‘no’ responses with an additional open category in which participants could manually report the subtype of supplementation. Autonomy-related variables were collected in the following categories: never, rarely, occasionally, often, or always. These categories were later collapsed into two categories: ‘never, rarely, or occasionally’ and ‘often or always’, representing a low and high degree of autonomy, respectively. Weight was measured twice in light clothing on an impedance scale (Tanita BC-418 MA, Arlington Heights, IL, USA) and recorded to the nearest 0.1 kg. Height was measured twice in an upright position with heels placed against the wall and with head fixed in Frankfurt plane using a wall-mounted stadiometer (Seca 222, Birmingham, UK) and recorded to the nearest 0.1 cm. Body mass index (BMI) was calculated using the standard formula. Blood samples were collected in the morning in 4-ml lithium-heparin-gel plasma tubes (Vacuette, Greiner Bio-one GmbH, Germany) after overnight fasting. The tubes were centrifuged at room temperature in a swing-out centrifuge at 1,500 g for 12 min and kept on ice until placement in a −80°C freezer. The samples were thawed in a refrigerator overnight before analyses of carotenoids and FAs were performed, at a laboratory with standardized procedures to perform such analyses.

### Assessment of carotenoids

Plasma (100 µL) was pipetted into vials, proteins were precipitated, and carotenoids were extracted with isopropanol containing an internal standard (β-Apo-8 carotenal). After thorough mixing and subsequent centrifugation, an aliquot of the isopropanol phase was injected into the HPLC-UV. Separations were performed on a 3-µm YMC C30 column (150×4.6 mm internal diameter; YMC, Kyoto, Japan). Analyses were performed on a 100-series HPLC with a 1,260 diode array detector (453 nm) (Agilent Technologies, Palo Alto, CA, USA).

### FA assessment

Erythrocytes were vortexed and pipetted into vials, and samples were methylated with 3N MeOH HCl. Methylated FAs were extracted with hexane, and the samples were neutralized with 3N KOH in water. The samples were then mixed and centrifuged, and the hexane phase was injected into a gas chromatograph with flame ionization detector. Analyses were performed on a 7890A GC with a split injector with a 7683B automatic liquid sampler using flame ionization detection (Agilent Technologies, Palo Alto, CA, USA). Separations were performed on an SP-2380 column (30 m×0.25 mm internal diameter×0.25 µm film thickness) from Supelco (Sigma-Aldrich, St. Louis, MO, USA).

### Statistics

Standard descriptive statistics were calculated for all variables. Initially chi-square, one-way analysis of variance (ANOVA), and independent *t*-test were used to identify differences between the subgroups with respect to background variables. Level of support was regarded as collider in the investigations comparing the different genetic subgroups, and was for this reason considered not relevant to adjust for in the statistical analysis. Normality was assessed, and when violated, log-transformed variables were used. Ordinal categorical variables were dichotomized to avoid zero-cell problems and loss of statistical power. Chi-square tests were used to investigate proportions with low and high intake frequencies of fish by diagnosis, due to a zero-cell. Otherwise logistic regression models adjusted for BMI were used to investigate the association between proportions with low- and high-frequency consumers in the different food categories and use of omega-3 supplementation by diagnosis. Linear regression adjusted for BMI was used to investigate plasma carotenoids and erythrocyte FAs to detect differences between diagnoses. Based on initial analysis by use of ANOVA with Tukey post hoc test, the PWS-group was used as reference and compared to a combined group of persons with DS and WS in comparison of plasma carotenoids. Similarly, in the investigation of erythrocyte FAs, WS was used as reference and compared to a combined group of persons with PWS and DS. Assumption of linearity, interaction, and standard residuals were investigated when relevant in the models. Chi-square tests were used to investigate differences in proportions with low and high food intake frequencies and degree of participation in food-related tasks based on BMI-category in the diagnoses. In these analyses, to ensure test validity, normal weight and overweight participants were collapsed into one group and compared to obese participants. Pearson's correlation test was used to assess the association between BMI and plasma carotenoids and erythrocyte FAs by diagnosis. Chi-square tests were used to investigate proportions with high- and low-frequency intake of foods, high and low degree of autonomy, when living in communities was compared with living with relatives for persons with DS. Similarly, Mann–Whitney tests were used to investigate differences in plasma carotenoids and erythrocyte omega-3 FAs when the living arrangements were compared. All statistical analyses were performed using SPSS 19 (SPSS Inc., Chicago, IL, USA), and *p*-values of less than 0.05 were regarded as statistically significant.

## Results

The characteristics of the study population are presented by diagnosis in Table 1. More females than males participated in the study; however, there was no significant difference in age or BMI between the sexes. Likewise, the sex distribution was similar in the genetic subgroups. Overweight and obesity were prevalent, and 78% of the total study population had a BMI >25 kg/m^2^. When the diagnosis-groups were compared, there was a small difference in BMI. Participants with DS and WS had the highest and lowest BMI's, respectively. Participants with DS living with their parents were on average 8.9 years younger than diagnosis-specific peers living in community settings. All persons with PWS followed a diet and had assistance to follow dietary regimens. None of the participants with PWS decided independently the amount of food to eat due to their well-known hyperphagia and high risk of developing obesity. However, variable degree of participation in food decisions was recorded. Some individuals did not take any part in decisions and preparation of food eaten, whereas most individuals took some part in food decisions for example, what type of fruit and vegetables to consume, what type of spreads to have on their bread, etc. Three persons with DS had celiac disease. Hypothyroidism was common and found in 17 persons with DS (43.6%), three persons with WS (12.0%), and three persons with PWS (13.6%).

**Table 1:** Characteristics of the study population by living arrangement and diagnosis

	Living in community residence			Living with relatives		
		
	Prader–Willi syndrome (*n*=20)	Williams syndrome (*n*=21)	Down syndrome (*n*=24)	Down syndrome (*n*=16)	*p* a Diagnosis	*p* b Living arrangements
Age (years)	29.3 (5.3)	33.6 (6.4)	30.4 (6.4)	21.5 (5.8)	0.067	<0.001**
Females (%)	55.0	66.7	62.5	62.5	0.739	1.000
BMI (kg/m^2^)	30.9 (6.1)	28.2 (5.8)	32.8 (6.6)	30.6 (6.3)	0.050	0.250
Smokers (%)	10.0	4.8	0	0	0.289	
Occupation					0.463	0.113
Student (%)	0	4.8	4.2	31.3		
Employed (%)	85.0	81.0	66.7	50.0		
Daycare attendant (%)	10.0	4.8	25.0	12.5		
Unemployed (%)	5.0	9.5	4.2	6.3		
Years lived in community residence (years)	8.5 (5.5)	9.4 (5.8)	7.3 (4.9)		0.443	
Level of support in community residence**					0.020*	
0–30 h/week (%)	40.0	47.6	54.2			
31–60 h/week (%)	10.0	23.8	20.8			
More than 60 h/week (%)	45.0	4.8	8.3			
Level of support unknown (%)	5.0	23.8	16.7			

The data are presented as percentage of the population or as the mean (standard deviation).

a *p*-Values are derived by comparison of persons in community residence with different diagnosis using chi-square tests and one-way analysis of variance (ANOVA)

b *p*-values are derived by comparison of persons with DS living in community residence to persons with DS living with relatives using chi-square tests and independent *t*-test.

* *p*<0.05.

** *p*<0.001.

**Fig. 1:**
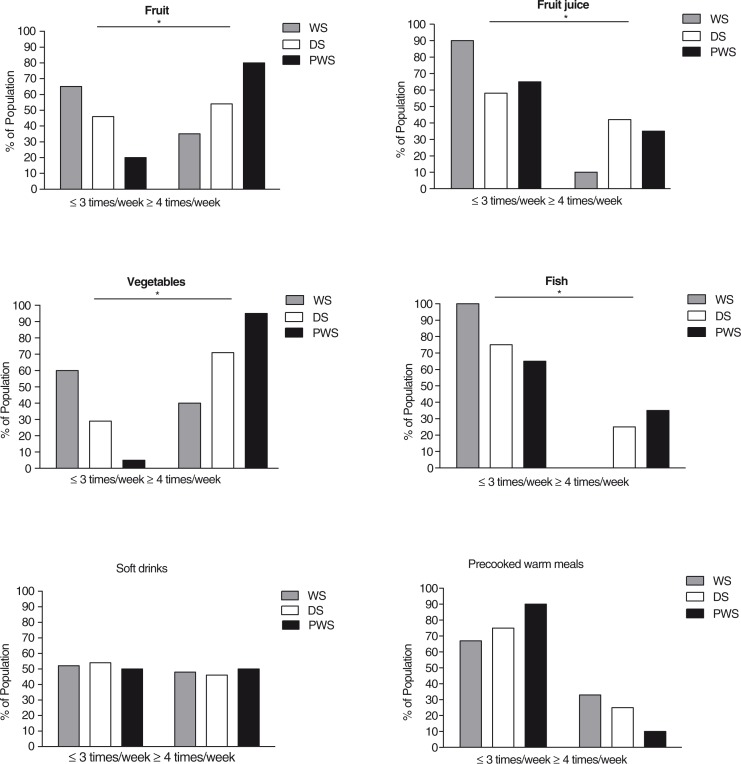
Proportion low- and high-frequency consumers by diagnosis. Intake frequencies are presented as a percentage of the population diagnosed with WS (*n*=21), DS (*n*=24) or PWS (*n*=20). *p<0.05 when participants with PWS were compared to participants with WS and DS using logistic regression models adjusted for BMI.

In the PWS-group, a total of 70, 25, and 85% of participants consumed daily fruit, fruit juice, and vegetables, respectively. In the DS-group daily consumption of fruit was recorded in 33%, fruit juice in 37%, and vegetables in 29%. For participants with WS, daily consumption of fruit, fruit juice, and vegetables was recorded as 15, 9, and 20%, respectively. The proportions with intake frequency of three times or less and four times or more a week (low- and high-frequency intake) by diagnosis are shown in Fig. 1. A larger proportion of participants with PWS were high-frequency consumers of fruit (*p*=0.012) and vegetables (potatoes excluded, *p*=0.004) compared to participants with WS and DS. Using participants with PWS as a reference in a logistic regression model adjusted for BMI, the odds ratio (OR) of being a high-frequency consumer of fruit was 0.10 (95% CI 0.02; 0.45) in the WS-group, and 0.32 (95% CI 0.08; 1.30) in the DS-group. Odds for being high-frequency consumers of vegetables were OR 0.03, (95% CI 0.003; 0.29) in the WS-group, and OR 0.14, (95% CI 0.02: 1.25) in the DS-group. The largest proportion of participants with high-frequency intake of fruit juice was in the DS-group. When compared to the PWS-group, we observed an OR of 1.35 (95% CI 0.39; 4.68) in the DS-group, and an OR of 0.19 (95% CI 0.03; 1.08) in the WS-group. Daily consumption of fish was reported in 25% of participants with PWS, 4% of participants with DS, and by none in the WS-group. A larger proportion of participants with WS were also found to be low-frequency consumers of fish (*p*=0.005) compared to participants with PWS and DS. Overall, 20% of participants with WS reported consuming fish less than once a week, whereas the numbers of participants with DS and PWS were 2 and 4%, respectively. In addition, the participants with WS were less likely to use omega-3 supplementation; 24% in the WS-group compared to 50% in the two other groups, respectively, OR 0.24 (95% CI 0.07; 0.82), *p*=0.023.

No significant differences were found among the groups with respect to low- and high-frequency intakes of soft drinks and precooked meals. The proportion of individuals consuming soft drinks daily or several times a day ranged from 21% in participants with DS to 31% in the PWS-group and 32% in the WS-group.

The plasma carotenoid concentrations and weight percent (w %) of omega-3 FAs in erythrocytes by diagnosis are shown in Table 2. In accordance with the results based on reported intake frequencies, participants with PWS were found to have higher plasma concentrations of total carotenoids compared with the other two groups, *p*<0.001. Compared to participants with DS and WS, participants with PWS also had higher plasma concentrations of the single carotenoids: lutein (*p*<0.001), zeaxanthin (*p*=0.006), β-cryptoxanthin (*p*<0.001), α-carotene (*p*<0.001), and β-carotene (*p*<0.001). No significant differences in these carotenoids were observed between participants with DS and WS.

**Table 2:** Carotenoids in plasma and omega-3 fatty acids in erythrocytes by diagnosis

	Living in community residence									Living with relatives		
		
	Prader–Willi syndrome (*n*=20)			Williams syndrome (*n*=21)			Down syndrome (*n*=24)			Down syndrome (*n*=16)		
				
Carotenoids (µmol/l)	Median	Q1	Q3	Median	Q1	Q3	Median	Q1	Q3	Median	Q1	Q3
Lycopene	0.806	0.564	0.918	0.579	0.490	0.875	0.810	0.554	1.113	0.822	0.686	0.896
Lutein	0.259*	0.220	0.328	0.149	0.119	0.267	0.186	0.145	0.206	0.178	0.143	0.211
Zeaxanthin	0.084*	0.051	0.104	0.051	0.029	0.091	0.054	0.048	0.076	0.060	0.042	0.068
β-Cryptoxanthin	0.258*	0.176	0.326	0.138	0.069	0.248	0.128	0.103	0.227	0.121	0.074	0.177
α-Carotene	0.227*	0.178	0.377	0.084	0.041	0.126	0.103	0.066	0.146	0.076	0.048	0.116
β-carotene	0.984*	0.519	1.335	0.456	0.327	0.538	0.351	0.239	0.482	0.365	0.262	0.465
Total carotenoids	2.76*	2.14	3.14	1.63	1.17	2.22	1.76	1.34	1.99	1.65	1.43	1.81
Omega-3 fatty acids (w %)												
ALA^a^	0.193	0.180	0.209	0.189	0.172	0.203	0.201	0.174	0.220	0.207	0.162	0.228
EPA^b^	2.00	1.30	2.83	1.00**	0.75	1.40	1.10	0.90	1.70	1.20	1.10	1.45
DPA^c^	2.60	2.40	3.05	2.50**	2.35	2.90	2.75	2.63	3.10	2.70	2.60	2.88
DHA^d^	6.90	6.00	8.03	5.40**	4.65	6.30	6.15	5.80	7.50	6.75	5.25	7.30
Total omega-3 FAs	12.12	9.80	13.50	9.30**	8.55	10.51	10.19	9.44	12.75	10.67	9.56	11.72

Median plasma concentrations of carotenoids and median erythrocyte fatty acids presented with 25th and 75th percentiles (Q1 and Q3).

a α-Linolenic acid, C18:3;

b eicosapentaenoic acid, C20:5;

c docosapentaenoic acid, C22:5;

d docosahexaenoic acid, C22:6.

* *p*<0.05 when participants with PWS were compared to participants with WS and DS using linear regression models, adjusted for BMI.

** *p*<0.05 when participants with WS were compared to participants with PWS and DS using linear regression models, adjusted for BMI.

When evaluating the omega-3 FAs in erythrocytes, lower levels of total omega-3 FAs were observed in the WS-group compared to the other two groups, *p*<0.001. The differences between the groups using WS as reference were also significant for the individual FAs: eicosapentaenoic acid (EPA) (*p*=0.002), docosapentaenoic acid (DPA) (*p*=0.001), and docosahexaenoic acid (DHA) (*p*<0.001). No association between erythrocyte omega-3 FAs and BMI was detected.

**Fig. 2:**
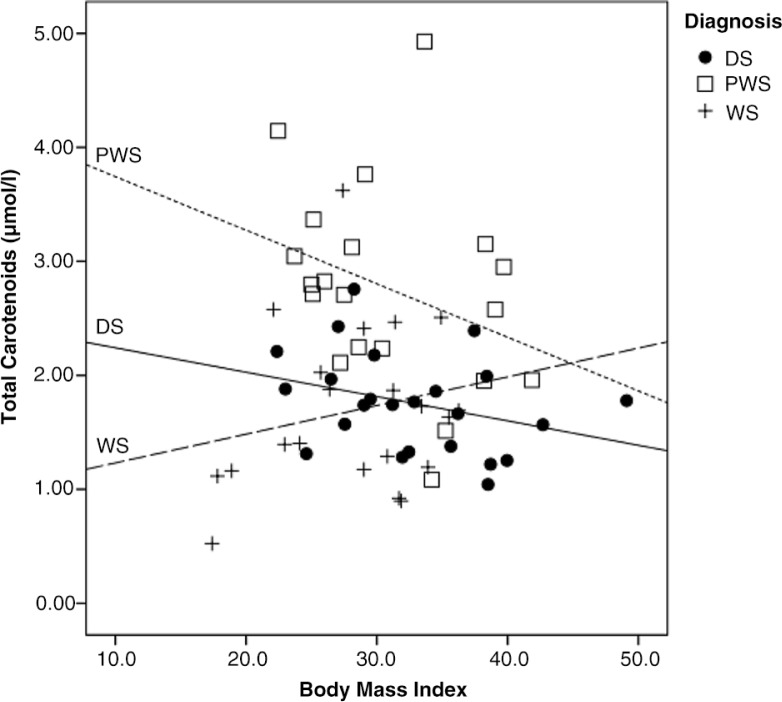
The association between BMI and total carotenoids by diagnosis. Scatter plot include participants with DS (*n*=40), participants with PWS (*n*=20), and participants with WS (*n*=21). The correlation was tested by use of person's correlation and significant for persons with DS, r−0.33, *p*=0.039.

Intake frequencies and participation in selection and preparation of foods was investigated in relation to diagnosis and BMI-category. In DS, normal weight and overweight participants were found to be more likely to consume fruits four times or more per week compared to obese participants, *p*=0.032. A similar tendency was observed for intakes of vegetables, *p*=0.081, whereas no other difference was observed in proportions with low and high intake frequencies of foods, or in participation in food selection and preparation by the groups based on BMI in DS. In PWS, normal weight and overweight participants reported more often intakes of fruit four times or more per week compared to obese participants, *p*=0.013. No other significant differences in the proportions with low and high intake frequencies of foods or degree of participation in food-related tasks were observed in the PWS-group when this was evaluated in BMI-based groups. This was also the case in the WS-group. In Fig. 2, the association between BMI and measured plasma carotenoids is shown in the different diagnose groups. Increased BMI was associated with a reduction in total plasma carotenoids for persons with PWS and DS. This correlation, however, was only significant in DS, r −0.33, *p*=0.039. In contrast, the lowest plasma carotenoid concentrations were found in those categorized as underweight and lower end of normal weight range among participants with WS.

**Fig. 3:**
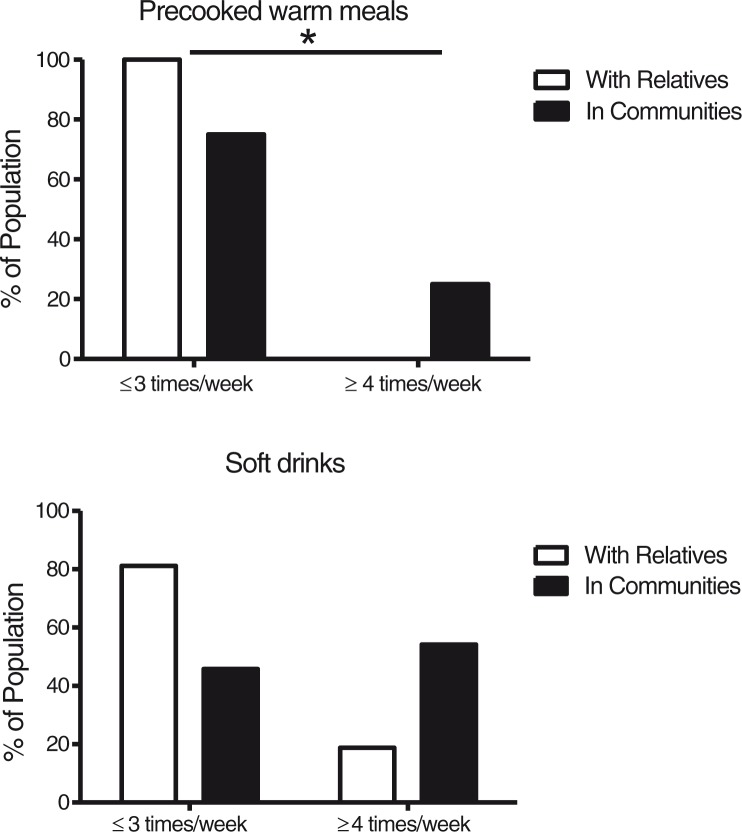
Proportion low- and high-frequency consumers based on living arrangement. Intake frequencies are presented as a percentage of the population with DS living with relatives (*n*=16) or in communities (*n*=24). *p<0.05 when participants with DS living in community residences were compared to diagnose specific peers living with relatives using chi-square tests.

**Fig. 4:**
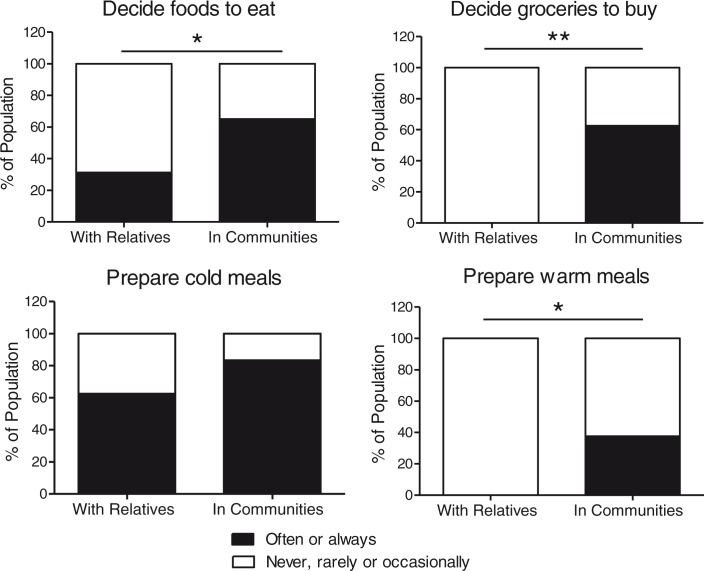
Food-related autonomy for persons with Down syndrome in different living arrangements. Degrees of participation are presented as a proportion (%) of the population living with relatives (*n*=16) or in communities (*n*=24). *p<0.05 when participants living in community residences were compared to participants living with relatives using chi-square tests. **p<0.001 when participants living in community residences were compared to participants living with relatives using chi-square tests.

When participants with DS living with parents (*n*=16) were compared to those living in communities (*n*=24), we found no differences in the proportions of low- and high-frequency consumers of fruit, fruit juice, vegetables, and fish. Moreover, no significant differences were observed in any of the biomarkers measured in blood from the two subgroups, as can be seen in Table 2. However, as shown in Fig. 3, a larger proportion of high-frequency consumers of precooked meals were observed in persons living in community residences (*p*=0.030). We also observed a trend toward a larger proportion of high-frequency consumers of soft drinks in community-dwelling participants (*p*=0.079). As displayed in Fig. 4, when compared to participants living with relatives, participants living in communities had a higher degree of participation in decisions regarding which foods to eat (*p*=0.037) and which groceries to buy (*p*<0.001), and participated more in the preparation of warm meals (*p*=0.005), whereas only small changes were observed between the living arrangements with respect to participation in cold meal preparation.

## Discussion

In this study, we describe the proportion with low- and high-frequency consumption of selected food groups that are of particular interest to health and obesity in community-dwelling participants with three different genetic conditions associated with ID and compare the results to individuals living with relatives. Norway has a general national policy and transition practice for persons with ID, and this provides a national cohort with reduced probability of systematic differences in level of functioning or comorbidities between the subgroups based on living arrangements. A difference in age between the persons living in community residence versus living with relatives was however seen, as people with ID living with relatives often moves to community residence before 25 years of age.

We included adolescents and adults in the study, and the participants themselves were used as informants. For this reason, the dietary assessment methods used were a very simplified electronic method to evaluate intake frequencies in selected food groups. The intake frequencies were recorded in quite wide categories and without estimation of portion sizes, or description of food group subtype consumed. This decision was based on limitations in cognitive abilities of the study populations, in combination with knowledge that intake frequency alone explains the major variation in intake (50). To supplement these self-reported intake data, biomarkers of dietary intakes were measured in blood. To the best of our knowledge, this is the first report to use objective biochemical markers to support the information about dietary intakes obtained from questionnaires in groups of adults associated with ID. Biomarkers have been shown to have relative good correlation with intake measured by other assessment techniques (27, 29, 31, 33). This is a strength in our study, given the methodological challenges in recording habitual diets in general, and the challenges in addressing diets in persons with ID living in community residences, where a significant proportion of the individuals live unsupervised for many hours a day. The latter is a methodological challenge, making the use of proxy reporters difficult.

Overweight and obesity was prevalent in the study population, and the mean BMI of 30.0 kg/m^2^ was elevated compared to the mean BMI of 27 kg/m^2^ reported from the general adult Norwegian population (51). Increased risk of overweight and obesity in adult populations with ID is well known and emphasizes the rationale for investigations of determinants, such as diets, that may contribute to the high risk of obesity in these populations.

In the general adult Norwegian population, daily consumption of fruit, fruit juice, and vegetables are reported in about 55, 40, and 60%, respectively (52). Thus, our data suggests larger proportion with daily consumption of fruit and vegetables in the PWS-group, and reduced proportion with daily consumption of fruit and vegetables in both the WS-group and the DS-group. Our data cannot be evaluated with respect to adherence to food based dietary guidelines for intakes of fruit and vegetables due to the types of methods used. However, intake frequencies of three times or less per week suggest intakes below the recommended level. In participants with WS, such low consumption frequency of fruit and vegetables was observed in 71 and 58%, respectively. In DS, 40% were low-frequency consumers of fruit and 25% were low-frequency consumers of vegetables, whereas the corresponding numbers for participants with PWS were 23 and 5%, respectively. These findings, with the exception of those in the PWS-group, were in accordance with the previously described low intakes of fruit and vegetables in several studies of community-dwelling persons with ID (15, 16, 18) and with DS (6). However, low intake frequency of fruit and vegetables has previously not been described for an adult population with WS. These findings are interesting in light of the high risk of developing hypertension associated with WS (53), and the fact that diets rich in fruits and vegetables may contribute to reduce the risk of hypertension (54). Even so, this association needs to be further evaluated in relation to WS.

The biomarkers studied overall support the data obtained from the food intake frequency questionnaire. A reduction in plasma carotenoids of about 40% in WS, and about 35% in DS, was observed when compared to measured levels in PWS. There are a few genetic alterations know to affect the level of carotenoids in plasma (55). However, none of these are located on the chromosomes known to be involved in the included subgroups, and provides support that the measured difference is due to consumption inequality. Unfortunately, the measured carotenoids cannot be used to calculate actual intake of fruit and vegetables in the groups. However, the underlying concept is that the use of such objective markers in blood is related to intake and takes in to account effect of absorption and metabolism such as oxidative stress and excretion, and by this provides supportive data that should be of high value in investigations on fruit and vegetable intakes and health outcomes (25).

Increased BMI was associated with a reduction in plasma carotenoid concentrations in the PWS and DS groups, although this correlation was only significant for individuals with DS. This might suggest that persons with increased BMI in some subgroups consume less fruit and vegetables. This tendency was supported by the results based on reported intake frequencies in relation to BMI-categories. This finding is furthermore in accordance with descriptions from other investigations in overweight and obese individuals with ID (16) and from adults in the general populations where BMI has been found to be negatively associated with plasma carotenoid levels (28). Even so, it has also been suggested that the observed difference in plasma carotenoids related to weight status also might be influenced by difference in metabolism of carotenoids in persons with excessive body weight compared to persons with normal weight, although, the possible impact of this currently is not well described (25). In WS, persons classified as having a BMI at the lower end of normal range or as being underweight, showed a tendency toward lower intake frequency of fruits and vegetables and lower plasma concentrations of carotenoids. No severely underweight individuals participated in the study, but these findings may indicate that feeding problems typically occurring in early childhood in this group (34), for some individuals may persist into adulthood and continue as selective eating or other eating disorders (56). Obesity is described to be the most frequent weight related problem in adults with ID (4, 5). Nevertheless, the diversity observed between the groups in this study elucidates the heterogeneity found in adults with ID who were also underweight, and that feeding problems are more frequently observed in these groups compared to the general population (4). Further studies investigating the association between diets and BMI should describe and take into account individuals with feeding problems and selective eating pattern, as these individuals might also be found among persons in the normal BMI-range.

About 10% of the general Norwegian population consumes seafood daily (52) and about 15% consume fish meals less than once a week (13). When the diagnosis-groups were compared, intake frequencies of fish and omega-3 supplementations were lowest in the WS population, and 20% of individuals in this group reported an intake frequency of less than once a week, which is below the recommended frequency of two to three times a week for prevention of secondary conditions (12). In WS, the combination of low-frequency consumption of fish, less use of omega-3 supplementation, and reduced level of long chained omega-3 FAs in erythrocyte membranes indicate increased risk of insufficient intakes of essential omega-3 FAs, and potentially also insufficient intake of vitamin D. In Norway, fish and omega-3 supplements containing vitamin D are the most important sources of vitamin D. The reduced intakes of fish and omega-3 supplements found in the WS-group may be due to their vitamin D content, where dietary restrictions in childhood, implemented to resolve sporadic hypercalcemia, may have contributed to a habit of low intakes that continue through adolescence and into adulthood.

Data from the present study indicate that participants with PWS comply better with dietary recommendations for fruit, vegetables, fish, and omega-3 intake than participants in the WS-group and, in some aspects, participants in the DS-group. We suspect that this difference may be explained by frequent use of restrictive low-energy diets, in combination with the descriptions of dietary vulnerabilities (41, 42), and increased level of support associated with PWS in our study population.

About one-third of the study population consumed soft drinks daily or several times a day, a result that is similar to what is described from the general population (52). Furthermore, there was a tendency toward higher intake of soft drinks in community-dwelling participants with DS compared to peers living with relatives. Frequent consumption of soft drinks among persons living in communities has also previously been reported (16, 18, 21). Sugar-sweetened soft drinks are important contributors to refined sugar intake (13) and are compared to artificially-sweetened beverages associated with less-favorable health outcome (57). Frequent use of sugar-sweetened soft drinks may be one of the underlying factors contributing to the increased risk of obesity in community-dwelling persons with ID (21). Unfortunately, in this study we do not have data to differentiate between the use of sugar-sweetened and artificially-sweetened soft drinks, and no association was observed between obesity and high intake frequency of soft drinks in this study. Nevertheless, daily use of soft drinks with and without sugar is in general associated with reduced dental health (58).

Intake of precooked meals was higher in community-dwelling participants with DS. The use of precooked meals in adults with ID can be a double-edged sword. On one hand, these meals can provide nutritional independence, but on the other hand, they are often energy dense, with high fat and salt content, and with low content of vegetables and fiber. Furthermore, they are often not adjusted to the reduced energy needs associated with DS and PWS, and if family-size package meals are selected, persons with ID eating alone might have difficulties dividing the meal into adequate portion size. For these reasons the total energy intake from and nutrient content of these meals needs to be evaluated, as our data indicate that they are an important part of the diet for a significant proportion of persons living in community residences. To comply with the law regulating the right to self-determination, the use of these meals also needs to be voluntary and not forced due to lack of proper training in cooking skills or assistance.

Living in communities was accompanied by increased influence on food choices and responsibility for foods. In this study, no difference in proportions with low and high intake frequencies of fruit, fruit juice, vegetables, fish, and omega-3 supplementation was detected when persons living in communities were compared to those living with relatives. This is an important finding as it is a common perception that increased autonomy in persons with ID in general leads to overall poor diets. Nevertheless, if individuals with ID are given freedom to make dietary decisions, this needs to be accompanied by proper training regarding healthy food choices and food preparation skills. Studies have shown that individuals with ID make healthier food choices after intervention targeting nutritional knowledge (59). Proper support in these aspects may also be of importance in the transition period when persons establish new routines in community settings. There might also be a need to increase knowledge and skills in food-related tasks among caregivers assisting these groups (60).

This study has several limitations. The method of recruitment and low participant numbers limit the statistical power and the generalizations, although approximately one-third of the known population of PWS and WS in Norway in the relevant age category participated, and we recruited participants from all over the country. No formal testing of intellectual functioning was conducted, and this is an important limitation, as there may be differences in these aspects related to the included genetic conditions, even though all three conditions are associated with similar level of ID. The fact that all participants cooperated well and were able to complete all aspects of the data collection, indicates that the average level of functioning in the study population might have been above the average in the respective diagnosis. The simplified dietary assessment technique used in this study does not include all aspects of dietary intake, and can only be used to describe some overall food intake frequency patterns. Additional use of digital photography of meals consumed would probably have improved the dietary intake estimates (61, 62). Furthermore, a validation of the individuals’ abilities to report diets at this consumption level would have been valuable. Nevertheless, an agreement in results between the reported intake frequencies and measured biomarkers was observed, providing credibility and support to the results obtained. For these reasons, the results of the present study must be regarded as explorative rather than conclusive.

Persons with WS had a less-favorable dietary pattern, as indicated by the larger proportion of low-frequency consumers of fruits and vegetables as well as lower plasma carotenoid concentrations when compared to persons with PWS. In addition, a larger proportion of persons with WS were low-frequency consumers of fish, fewer used omega-3 supplementation, and lower concentrations of erythrocyte omega-3 FAs were observed in persons with WS compared to persons with PWS and DS. A more comprehensive assessment of diets in adult persons with WS is warranted and should include assessment of medical and physical aspects that might influence food intakes. An increased proportion of persons with DS living in communities were high-frequency consumers of precooked meals, and there was a tendency toward a larger proportion of high-frequency consumers of soft drinks, when compared to peers living with relatives. Apart from this, their intake frequencies of the investigated foods were similar to those living with relatives, even though they had higher degree of food-related autonomy.

## References

[1] Englund A, Jonsson B, Zander CS, Gustafsson J, Anneren G (2013). Changes in mortality and causes of death in the Swedish Down syndrome population. Am J Med Genet A.

[2] Zhu JL, Hasle H, Correa A, Schendel D, Friedman JM, Olsen J (2013). Survival among people with Down syndrome: a nationwide population-based study in Denmark. Genet Med.

[3] Beadle-Brown J, Mansell J, Kozma A (2007). Deinstitutionalization in intellectual disabilities. Curr Opin Psychiatry.

[4] Hove O (2004). Weight survey on adult persons with mental retardation living in the community. Res Dev Disabil.

[5] De Winter CF, Bastiaanse LP, Hilgenkamp TI, Evenhuis HM, Echteld MA (2012). Overweight and obesity in older people with intellectual disability. Res Dev Disabil.

[6] Braunschweig CL, Gomez S, Sheean P, Tomey KM, Rimmer J, Heller T (2004). Nutritional status and risk factors for chronic disease in urban-dwelling adults with Down syndrome. Am J Ment Retard.

[7] Robertson J, Emerson E, Gregory N, Hatto C, Turner S, Kessissoglou S (2000). Lifestyle related risk factors for poor health in residential settings for people with intellectual disabilities. Res Dev Disabil.

[8] World Cancer Research Fund/American Institute of Cancer Research (2007). Food, nutrition, physical activity, and the prevention of cancer: a global perspective.

[9] World Health Organization (2003). Diet, nutrition, and the prevention of chronic diseases: report of a joint WHO/FAO expert consultation.

[10] Dauchet L, Amouyel P, Hercberg S, Dallongeville J (2006). Fruit and vegetable consumption and risk of coronary heart disease: a meta-analysis of cohort studies. J Nutr.

[11] Mozaffarian D, Wu JH (2011). Omega-3 fatty acids and cardiovascular disease: effects on risk factors, molecular pathways, and clinical events. J Am Coll Cardiol.

[12] Norwegian National Nutrition Council (2011). Dietary recommendations for promotion of public health and prevention of chronic diseases. Methiology and scientific evidence.

[13] Totland TH, Melnæs BK, Lundberg-Hallén N, Helland-Kigen KM, Lund-Blix NA, Myhre JB (2012). Norkost 3: a national dietary survey in Norway among men and women 18–70 years of age.

[14] Brustad M, Braaten T, Lund E (2004). Predictors for cod-liver oil supplement use – the Norwegian Women and Cancer Study. Eur J Clin Nutr.

[15] Draheim CC, Stanish HI, Williams DP, McCubbin JA (2007). Dietary intake of adults with mental retardation who reside in community settings. Am J Ment Retard.

[16] Ptomey L, Goetz J, Lee J, Donnelly J, Sullivan D (2013). Diet quality of overweight and obese adults with intellectual and developmental disabilities as measured by the healthy eating index-2005. J Dev Phys Disabil.

[17] de Winter CF, Magilsen KW, van Alfen JC, Penning C, Evenhuis HM (2009). Prevalence of cardiovascular risk factors in older people with intellectual disability. Am J Intellect Dev Disabil.

[18] Adolfsson P, Sydner YM, Fjellstrom C, Lewin B, Andersson A (2008). Observed dietary intake in adults with intellectual disability living in the community. Food Nutr Res.

[19] Soler Marin A, Xandri Graupera JM (2011). Nutritional status of intellectual disabled persons with Down syndrome. Nutr Hosp.

[20] Vartanian LR, Schwartz MB, Brownell KD (2007). Effects of soft drink consumption on nutrition and health: a systematic review and meta-analysis. Am J Public Health.

[21] Hsieh K, Rimmer JH, Heller T (2014). Obesity and associated factors in adults with intellectual disability. J Intellect Disabil Res.

[22] Johnson C, Hobson S, Garcia AC, Matthews J (2011). Nutrition and food skills education for adults with developmental disabilities. Can J Diet Pract Res.

[23] Mesas AE, Guallar-Castillon P, Leon-Munoz LM, Graciani A, Lopez-Garcia E, Gutierrez-Fisac JL (2012). Obesity-related eating behaviors are associated with low physical activity and poor diet quality in Spain. J Nutr.

[24] Humphries K, Traci MA, Seekins T (2009). Nutrition and adults with intellectual or developmental disabilities: systematic literature review results. Intellect Dev Disabil.

[25] Jenab M, Slimani N, Bictash M, Ferrari P, Bingham SA (2009). Biomarkers in nutritional epidemiology: applications, needs and new horizons. Hum Genet.

[26] Potischman N (2003). Biologic and methodologic issues for nutritional biomarkers. J Nutr.

[27] Carlsen MH, Karlsen A, Lillegaard IT, Gran JM, Drevon CA, Blomhoff R (2011). Relative validity of fruit and vegetable intake estimated from an FFQ, using carotenoid and flavonoid biomarkers and the method of triads. Br J Nutr.

[28] Al-Delaimy WK, van Kappel AL, Ferrari P, Slimani N, Steghens JP, Bingham S (2004). Plasma levels of six carotenoids in nine European countries: report from the European Prospective Investigation into Cancer and Nutrition (EPIC). Public Health Nutr.

[29] Brevik A, Andersen LF, Karlsen A, Trygg KU, Blomhoff R, Drevon CA (2004). Six carotenoids in plasma used to assess recommended intake of fruits and vegetables in a controlled feeding study. Eur J Clin Nutr.

[30] Hodson L, Skeaff CM, Fielding BA (2008). Fatty acid composition of adipose tissue and blood in humans and its use as a biomarker of dietary intake. Prog Lipid Res.

[31] Takkunen M, Agren J, Kuusisto J, Laakso M, Uusitupa M, Schwab U (2013). Dietary fat in relation to erythrocyte fatty acid composition in men. Lipids.

[32] Fekete K, Marosvolgyi T, Jakobik V, Decsi T (2009). Methods of assessment of n-3 long-chain polyunsaturated fatty acid status in humans: a systematic review. Am J Clin Nutr.

[33] Sun Q, Ma J, Campos H, Hankinson SE, Hu FB (2007). Comparison between plasma and erythrocyte fatty acid content as biomarkers of fatty acid intake in US women. Am J Clin Nutr.

[34] Morris CA (2010). Introduction: Williams syndrome. Am J Med Genet C Semin Med Genet.

[35] Hattori M, Fujiyama A, Taylor TD, Watanabe H, Yada T, Park HS (2000). The DNA sequence of human chromosome 21. Nature.

[36] Cassidy SB, Schwartz S, Miller JL, Driscoll DJ (2012). Prader-Willi syndrome. Genet Med.

[37] Melville CA, Cooper SA, McGrother CW, Thorp CF, Collacott R (2005). Obesity in adults with Down syndrome: a case-control study. J Intellect Disabil Res.

[38] Holland AJ, Treasure J, Coskeran P, Dallow J, Milton N, Hillhouse E (1993). Measurement of excessive appetite and metabolic changes in Prader-Willi syndrome. Int J Obes Relat Metab Disord.

[39] Butler MG, Theodoro MF, Bittel DC, Donnelly JE (2007). Energy expenditure and physical activity in Prader-Willi syndrome: comparison with obese subjects. Am J Med Genet A.

[40] State MW, Dykens EM, Rosner B, Martin A, King BH (1999). Obsessive-compulsive symptoms in Prader-Willi and “Prader-Willi-Like” patients. J Am Acad Child Adolesc Psychiatry.

[41] Miller JL, Lynn CH, Shuster J, Driscoll DJ (2013). A reduced-energy intake, well-balanced diet improves weight control in children with Prader-Willi syndrome. J Hum Nutr Diet.

[42] Goldstone AP, Holland AJ, Hauffa BP, Hokken-Koelega AC, Tauber M (2008). speakers contributors at the Second Expert Meeting of the Comprehensive Care of Patients with PWS. Recommendations for the diagnosis and management of Prader-Willi syndrome. J Clin Endocrinol Metab.

[43] Butler MG (2006). Management of obesity in Prader-Willi syndrome. Nat Clin Pract Endocrinol Metab.

[44] Phillips AC, Holland AJ (2011). Assessment of objectively measured physical activity levels in individuals with intellectual disabilities with and without Down's syndrome. PLoS One.

[45] Temple VA, Stanish HI (2009). Pedometer-measured physical activity of adults with intellectual disability: predicting weekly step counts. Am J Intellect Dev Disabil.

[46] Luke A, Sutton M, Schoeller DA, Roizen NJ (1996). Nutrient intake and obesity in prepubescent children with Down syndrome. J Am Diet Assoc.

[47] Murray J, Ryan-Krause P (2010). Obesity in children with Down syndrome: background and recommendations for management. Pediatr Nurs.

[48] Holm VA, Cassidy SB, Butler MG, Hanchett JM, Greenswag LR, Whitman BY (1993). Prader-Willi syndrome: consensus diagnostic criteria. Pediatrics.

[49] Preus M (1984). The Williams syndrome: objective definition and diagnosis. Clin Genet.

[50] Noethlings U, Hoffmann K, Bergmann MM, Boeing H (2003). European Investigation into Cancer and Nutrition. Portion size adds limited information on variance in food intake of participants in the EPIC-Potsdam study. J Nutr.

[51] Holmen TH, Langhammer A, Midthjell K, Krokstad S (2011). Somatic health. Public health development, the hunt study, Norway.

[52] Statistics Norway (2013). Health, care and social relations, survey on living conditions, 2012. Statistics Norway.

[53] Adams GN, Schmaier AH (2012). The Williams-Beuren Syndrome-a window into genetic variants leading to the development of cardiovascular disease. PLoS Genet.

[54] Wang L, Manson JE, Gaziano JM, Buring JE, Sesso HD (2012). Fruit and vegetable intake and the risk of hypertension in middle-aged and older women. Am J Hypertens.

[55] Borel P, Moussa M, Reboul E, Lyan B, Defoort C, Vincent-Baudry S (2007). Human plasma levels of vitamin E and carotenoids are associated with genetic polymorphisms in genes involved in lipid metabolism. J Nutr.

[56] Young T, Apfeldorf W, Knepper J, Yager J (2009). Severe eating disorder in a 28-year-old man with William's syndrome. Am J Psychiatry.

[57] de Koning L, Malik VS, Rimm EB, Willett WC, Hu FB (2011). Sugar-sweetened and artificially sweetened beverage consumption and risk of type 2 diabetes in men. Am J Clin Nutr.

[58] Tahmassebi JF, Duggal MS, Malik-Kotru G, Curzon ME (2006). Soft drinks and dental health: a review of the current literature. J Dent.

[59] Mann J, Zhou H, McDermott S, Poston MB (2006). Healthy behavior change of adults with mental retardation: attendance in a health promotion program. Am J Ment Retard.

[60] Melville CA, Hamilton S, Miller S, Boyle S, Robinson N, Pert C (2009). Carer knowledge and perceptions of healthy lifestyles for adults with intellectual disabilities. J Appl Res Intellect Disabil.

[61] Ptomey LT, Willis EA, Goetz JR, Lee J, Sullivan DK, Donnelly JE (2015). Digital photography improves estimates of dietary intake in adolescents with intellectual and developmental disabilities. Disabil Health J.

[62] Humphries K, Traci MA, Seekins T (2008). Food on film: pilot test of an innovative method for recording food intake of adults with intellectual disabilities living in the community. J Appl Res Intellect Disabil.

